# Risk of Stroke in Migraineurs Using Triptans. Associations with Age, Sex, Stroke Severity and Subtype

**DOI:** 10.1016/j.ebiom.2016.02.039

**Published:** 2016-02-27

**Authors:** Vanna Albieri, Tom Skyhøj Olsen, Klaus Kaae Andersen

**Affiliations:** a Statistics, Bioinformatics and Registry, Danish Cancer Society Research Center, 2100 Copenhagen, Denmark; b Department of Neurology, Bispebjerg University Hospital, 2400 Copenhagen, Denmark

**Keywords:** Stroke, Ischemic stroke, Hemorrhagic stroke, Migraine, Triptans

## Abstract

**Background:**

Identifying migraineurs by triptan utilization we studied risk for stroke in migraineurs compared to the general population.

**Methods:**

A cohort study including all citizens 25–80 years of age in Denmark 2003–2011 was conducted. All persons prescribed triptans, and all those hospitalized for a first stroke were identified in the Danish Registries. Information on stroke severity/subtype and cardiovascular risk factors was available for stroke patients.

**Findings:**

Of the 49,711 patients hospitalized for a first stroke, 1084 were migraineurs using triptans. Adjusting for age, sex, income, and educational level, risk for stroke was higher among migraineurs in respect to all strokes (RR 1.07; CI 1.01–1.14) and ischemic strokes (RR 1.07; CI 1.00–1.14). Risk for hemorrhagic stroke was increased but only in women (RR 1.41; CI 1.11–1.79). Risk was for mild strokes (RR 1.31; CI 1.16–1.48) while risk for severe strokes was lower among migraineurs (RR 0.77; CI 0.65–0.91). Risk was age-related; highest among women 25–45 years (RR ≈ 1.7). Risk was unrelated to numbers of dispensations.

**Interpretation:**

Migraineurs identified by triptan utilization had higher risk for stroke. Strokes were minor and cardiovascular risk factors were less prevalent pointing to a migraine-specific etiology of stroke different from that of thromboembolism.

## Highlights

• Migraineurs (identified by triptan utilization) are at higher risk for stroke.
• The risk is not related to use of triptans as such. Rather the risk seems to be a consequence of the underlying migraine disease.
• In migraineurs strokes are minor and less often associated classical stroke risk factor*s. Major* strokes are uncommon.
• The study points to an etiology of stroke in migraine different from thromboembolism, probably at the microvascular level.

We used triptan utilization as an alternative way of identifying migraine. In this study of the entire Danish population aged 25–80 years migraineurs identified in this way were at higher risk for stroke. The increased risk was not related to use of triptans as such: Rather the risk seems to be a consequence of the underlying migraine disease. Characteristically, strokes in migraineurs are minor strokes and less often associated with classical stroke risk factors. Major strokes are uncommon. The study points to an etiology of stroke in migraineurs different from that of atherosclerotic thromboembolism, probably at the microvascular level.

## 1 Introduction

Migraine is associated with a two-fold increased relative risk for stroke (Etminan et al., 2005, Schurks et al., 2009, Spector et al., 2010). Etiology of stroke in migraine remains still obscure (Kurth and Diener, 2012). It is not known whether it is thromboembolic or migraine-specific being different from that of thromboembolism (Kurth and Diener, 2012). Studies relate migraine to hemorrhagic stroke but they are too few and too small to make conclusions about etiology (Sacco et al., 2013, Kurth and Tzourio, 2013).

The reason for our insufficient knowledge on stroke in migraineurs is in part that the increased risk in absolute terms is small and available studies on the risk for stroke among migraineurs are hampered by small numbers weakening the reliability of risk estimations (Kurth and Diener, 2012).

As an alternative way of establishing a sizeable cohort of migraine patients with stroke we identified migraine through triptan utilization. Based on this cohort the purpose of this study was to estimate risk of stroke and to characterize strokes by age, sex, subtype and severity in the population of Danes with migraine who had been prescribed triptans.

## 2 Methods

The design of this study was based on the idea of establishing a large cohort of migraine patients identified by triptan utilization.

The study is a cohort study on all Danes aged 25–80 years who lived in Denmark during some or all of the period between January 1, 2003 and December 31, 2011. The cohort was linked to the Danish registries by the unique personal identification number to obtain information on triptan use, strokes and confounders (i.e. education and disposable income).

In Denmark triptans need prescription by a physician. Information on prescriptions for triptans (ATC-code N02CC) and numbers of prescriptions dispensed was obtained from the Danish Registry of Prescriptions. In Denmark triptans cannot be dispensed without prescription by an authorized physician. A prescription is reported in the Danish Registry of Prescriptions only if the patient buys the prescribed medication in any pharmacy in Denmark.

Information on hospitalization for stroke was obtained from the Danish Stroke Registry (Olsen et al., 2007, Mainz et al., 2004). Stroke was defined according to World Health Organization criteria (Report of the WHO Task Force on Stroke and other Cerebrovascular Disorders, 1989) and we included incident hospital admissions for first-ever stroke (ischemic or hemorrhagic; ICD-10 codes I61 and I63) in the period 2003–2011. For patients with multiple hospital admissions, only the first admission was included. Transient ischemic attacks were not included in the Registry. Patients aged < 25 and > 80 years were excluded from the study, as well as patients for whom scanning was not performed (0.4%)/result not available (0.7%). Most stroke patients (90%) are admitted to hospital, as access to hospital care is free in Denmark (Jørgensen et al., 1992). Information on education and disposable income for the cohort was obtained from Statistics Denmark (Dalton et al., 2008); both variables are associated with incidence of stroke and to some level proxies for lifestyle factors such as smoking (Dalton et al., 2008). Education was grouped into three categories: basic/high school (7–12 years of primary, secondary, and grammar-school education); vocational (10–12 years of education including vocational training); and higher (≥ 13 years of education) (Dalton et al., 2008). People for whom information on education was missing were excluded (14%). Disposable income was defined as household income after taxation and interest per person, adjusted for the number of people in the household and deflated according to the 2000 value of the Danish crown (DKK). For the analyses, disposable income was categorized into the 20th, 40th, 60th, and 80th percentiles of the age and gender-specific income distribution.

To study the association between migraine (identified through triptan utilization) and the risk factor profile among stroke patients, we included information from Danish Stroke Registry. These data include age, sex, stroke severity on admission measured on the Scandinavian stroke scale (Lindenstrøm et al., 1991), stroke subtype (ischemic/hemorrhagic) and cardiovascular profile. The Scandinavian stroke scale is a validated neurological scale of stroke severity from 0 to 58, lower scores indicating more severe strokes (Lindenstrøm et al., 1991). Ischemic stroke was distinguished from hemorrhagic stroke by computed tomography or magnetic resonance scanning. The cardiovascular profile (only available for the stroke patients) included information on alcohol consumption (under/over the limit set by the Danish National Board of Health) and current daily smoking. Diabetes mellitus, atrial fibrillation (chronic or paroxystic), arterial hypertension, previous myocardial infarction, previous stroke, and intermittent arterial claudication were diagnosed according to current Danish standards (Mainz et al., 2004).

The study protocol was approved by the board of the Danish Stroke Registry and the Danish Data Protection Agency (journal number 2012-41-0719).

### 2.1 Statistics

We conducted a prospective analysis of the entire Danish population aged 25–80 in the period 2003–2011 and estimated the association between migraine identified through triptan utilization and risk of hospitalization for stroke. The cohort was tabulated in grouped-data format, so that data were collapsed into strata, with one stratum for each combination of the available covariates (i.e. sex, age, calendar year, education, disposable income, and triptan use). Each stratum provided the total number of strokes and total follow-up time (person-year) at risk. Associations between migraine identified through triptan utilization and stroke was estimated with Poisson regression models (McCullagh and Nelder, 1989) and effect estimates are presented as relative risks (RRs) with 95% confidence intervals (CIs). Triptan use was calculated from the number of packages prescribed during the five years before the stroke and was then categorized into: no use and use of at least one package in the previous five years. The models were adjusted for income and education the year before the stroke, calendar year, sex, and age (modeled as a restricted cubic spline with four knots) the year of the stroke. Following the overall analysis, subanalyses were undertaken considering subtypes of strokes as outcome, i.e. ischemic strokes, hemorrhagic strokes and stroke severity categorized by quartile on the Scandinavian scale (0–37, 38–50, 51–56, 57–58). As sensitivity analyses we examined the effect of triptan use within one-year, two-years, three-years, and four-years time window prior to index data and evaluated triptan use the five years before stroke with the additional categorization: no use, use of only a package, and use of two and more packages. Furthermore, we examined the dose–response relationship between number of prescriptions dispensed within the five-years and one-year time window as a continuous and linear variable on the log incidence rate.

The possible effect modification of triptan use by age was evaluated by testing the interaction between age and triptan use by means of likelihood ratio tests. The effect by age was then inspected visually for ease of interpretation. We also evaluated the effect modification in the risk of stroke in migraine identified through triptan utilization with respect to ischemic stroke vs. hemorrhagic stroke and among stroke severity categories.

We made additional subanalysis including only women between 25 and 50 years in order to study effect modification by oral contraceptive use (ATC-code G03A). The subanalysis was conducted by means of Cox regression including use of oral contraception in addition to the other adjustment variables in the main analysis. Oral contraceptive use was defined as ever/never use within each calendar year and allowed to change status as time was progressing.

Finally, we described prevalence of risk factors in stroke patients by triptan use. Differences in risk factor profile between migraine patients identified through triptan utilization and non-users of triptans were evaluated by the inverse probability weights method to adjust for age at stroke followed by Wald test (Cole and Hernán, 2004). All analyses were conducted with R statistical software (R Core Team, 2013), and 0.05 was considered to represent statistical significance.

## 3 Results

The participants contributed 32.923 million person–years to the analysis; of these, triptan-users contributed 1.105 million person–years. During the study period, 49,711 Danes aged 25–80 years were registered in the Danish Stroke Register as having been hospitalized for a first stroke. Among these, 1084 had been prescribed triptans within the past five-years prior to index date. The mean number of prescriptions dispensed during five years was 22 packages (Inter quartile range (IQR) 24 packages; 1st quartile: 4 packages; 3 quartile 28 packages).

Demographic data for users and non-users of triptans are presented in Table 1. Triptan users were younger, more often women, and more often belonged to low-income and short-education groups. The crude incidence of hospitalization for stroke was 1.51 per 1000 per year for non-users of triptans and 1.36 per 1000 per year for users.

**Table 1:** Basic characteristics of the Danish population aged 25–80 years in the period 2003–2011 by triptan use.

		Non-users				Users			
		%^a^	10^5^ person-years	No. of events	Incidence 10^2^ person-years^b^	%^a^	10^5^ person-years	No. of events	Incidence 10^2^ person-years^b^
All			318.18	48,627	1.51		11.05	1084	1.36
Sex	Male	50.77	161.51	28,676	1.84	20.54	2.27	328	1.83
	Female	49.23	156.67	19,951	1.20	79.46	8.78	756	1.22
Income (quintile)	1st	19.9	63.29	11,594	1.84	18.42	2.03	264	1.77
	2nd	20.04	63.75	11,083	1.72	19.53	2.16	225	1.45
	3rd	20.04	63.78	9754	1.51	19.82	2.19	214	1.50
	4th	20.03	63.75	8653	1.34	20.46	2.26	196	1.30
	5th	19.99	63.64	7543	1.17	21.77	2.41	185	0.95
Education	Basic	10.74	34.20	1797	1.53	12.18	1.35	90	0.41
	Vocational	61.08	194.28	38,468	1.17	55.56	6.14	741	1.07
	Higher	25.56	81.43	7178	1.63	30.51	3.37	232	1.43
	Unknown	2.62	8.33	1184	1.58	1.75	0.19	21	1.44
Age (years)	≤ 35	20.79	66.30	598	0.09	18.92	2.09	35	0.17
	36–47	26.07	83.10	3110	0.37	33.87	3.74	209	0.56
	48–61	27.98	89.08	12,223	1.37	34.08	3.77	434	1.15
	≥ 62	25.16	79.70	32,696	4.10	13.13	1.45	406	2.81

a Percentages of non-users and users of triptan in the total population.

b Age-adjusted incidence with direct standardization by use of the whole Danish cohort as the reference population.

**Fig. 1:**
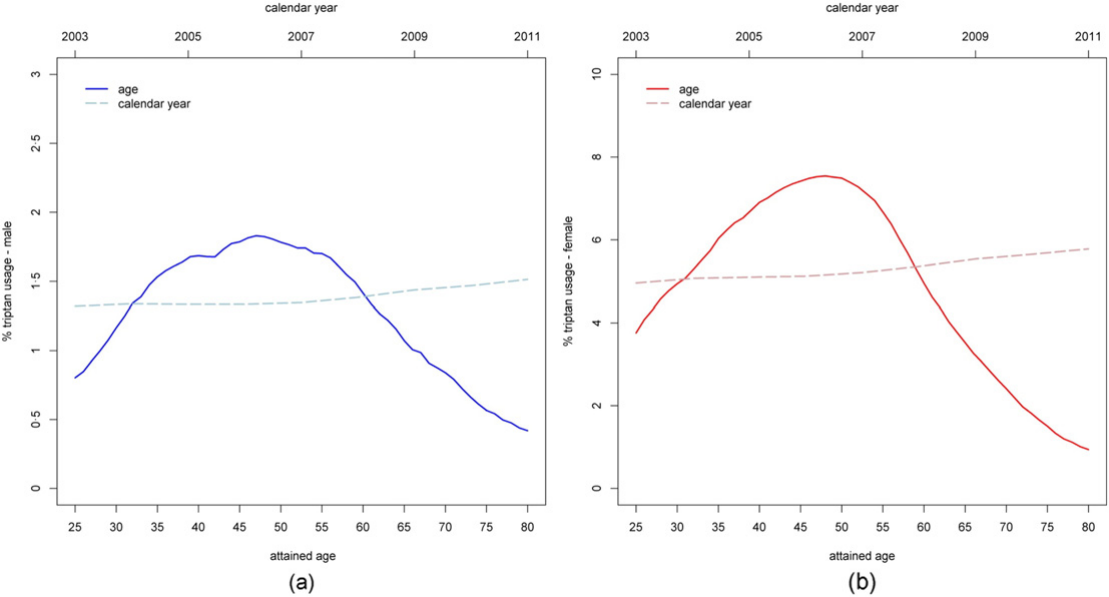
Percentages of triptan users by attained age and calendar year in the Danish population aged 25–80 years in the period 2003–2011; (a) males, (b) females.

The relation between age, sex and use of triptans is illustrated in Fig. 1. Triptans were prescribed to 5% of women and 1.4% of men aged 25–80 years during the study period. Usage reached a maximum at the age of 45–50 years (7·5% of women and 1·8% of men) and decreased rapidly with age after the end of the fifties.

The relative risks for stroke among migraine patients identified through triptan utilization are shown in Table 2. The risk was increased for all strokes (RR 1.07; CI 1.01–1.14) and ischemic strokes (RR 1.07; CI 1.00–1.14) while not for hemorrhagic strokes (RR 1.18; CI 0·95–1.45). Stratifying by sex the risk for all stroke and ischemic stroke was increased in both sexes There was an increased risk for hemorrhagic stroke in women (RR 1.41; CI 1.11–1.80) but not in men. Test for effect modification in risk of stroke with respect to triptan use for ischemic versus hemorrhagic stroke showed no statistical significant difference (Table 2).

**Table 2:** Relative risks (RRs) and 95% confidence intervals (CIs) for triptan users versus non-users by stroke type in the complete data.

Stroke	Triptan	General		Male		Female	
		No. of events (10^5^ person-years)	RR^a^ (CI)	No. of events (10^5^ person-years)	RR^b^ (CI)	No. of events (10^5^ person-years)	RR^b^ (CI)
All	Non-users	48,627 (318.18)	Ref.	28,676 (161.51)	Ref.	19,951 (156.67)	Ref.
	Users	1084 (11.05)	1.07 (1.01–1.14)	328 (2.27)	1.06 (0.95–1.19)	756 (8.78)	1.10 (1.02–1.18)
Ischemic	Non-users	44,852 (318.18)	Ref.	26,467 (161.51)	Ref.	18,385 (156.67)	Ref.
	Users	993 (11.05)	1.07 (1.00–1.14)	310 (2.27)	1.09 (0.97–1.22)	683 (8.78)	1.07 (0.99–1.16)
Hemorrhagic	Non-users	3775 (318.18)	Ref.	2209 (161.51)	Ref.	1566 (156.67)	Ref.
	Users	91 (11.05)	1.18 (0.95–1.45)	18 (2.27)	0.76 (0.48–1.21)	73 (8.78)	1.41 (1.11–1.80)
p-value†			0.44		0.11		0.08
Severity score							
0–37	Non-users	9105 (318.18)	Ref.	4971 (161.51)	Ref.	4134 (156.67)	Ref.
	Users	145 (11.05)	0.71 (0.65–0.91)	40 (2.27)	0.75 (0.55–1.02)	105 (8.78)	0.73 (0.61–0.89)
38–50	Non-users	10,244 (318.18)	Ref.	5994 (161.51)	Ref.	4250 (156.67)	Ref.
	Users	201 (11.05)	0.95 (0.83–1.09)	65 (2.27)	1.01 (0.79–1.29)	136 (8.78)	0.93 (0.78–1.10)
51–56	Non-users	13,777 (318.18)	Ref.	8371 (161.51)	Ref.	5406 (156.67)	Ref.
	Users	342 (11.05)	1.20 (1.08–1.34)	110 (2.27)	1.22 (1.01–1.48)	232 (8.78)	1.24 (1.09–1.41)
57–58	Non-users	10,064 (318.18)	Ref.	6254 (161.51)	Ref.	3810 (156.67)	Ref.
	Users	272 (11.05)	1.31 (1.16–1.48)	83 (2.27)	1.24 (1.00–1.54)	189 (8.78)	1.44 (1.24–1.66)
p-value†			< 0.001		0.02		< 0.001

a Adjusted for sex, income, and education the year before the stroke and for age (restricted cubic spline, four knots) and calendar year the year of stroke.

b Adjusted for income and education the year before the stroke and for age (restricted cubic spline, four knots) and calendar year the year of stroke.

† Test for effect modification in risk of stroke with respect to triptan use, i.e. ischemic stroke VS hemorrhagic stroke and among stroke severity categories.

**Fig. 2:**
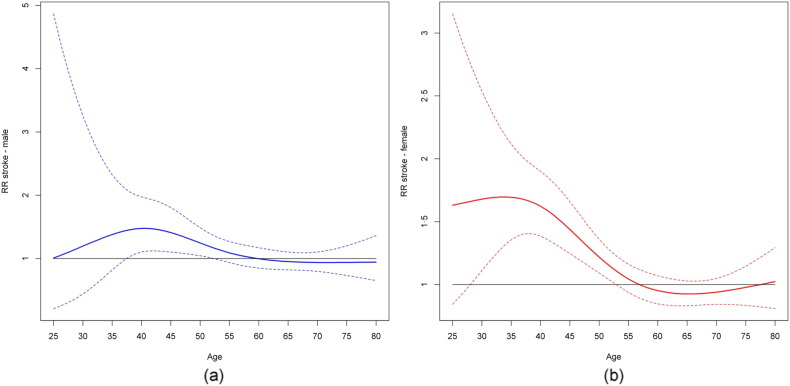
Relative risks and confidence intervals for all stroke types for the interaction between triptan (users versus non-users) and age modeled with restricted cubic spline. For male population (a), *p*-value for interaction is 0·073; for female population (b), *p*-value for interaction is < 0.0001.

Overall the risk for severe stroke was lower among migraine patients identified through triptan utilization (RR 0.71; CI 0.65–0.91), while the risk for mild stroke was higher (RR 1.31; CI 1.16–1.48). The relation between risk for stroke by age in migraine patients identified through triptan use is shown in Fig. 2. The estimated risk was closely associated with age and was highest among women aged 25–45 years (RR ≈ 1.7), decreasing rapidly thereafter with no association with triptan use after the age of about 55 years.

As a sensitivity analysis we examined the effect of triptan use within one-year, two-years, three-years, and four-years time window prior to index data. We found virtually no difference in effect estimates between the analysis defining exposure in one and five years' time. Also the additional categorization for triptan use (no use, use of one package, and use of two and more packages) did not show different results in respect to the main analysis. We examined the dose response effect by including the number of prescriptions as a continuous variable along with the indicator of triptan use. This parametrization of the model allowed for estimation of RR per prescription within triptan users. Overall, we find the RR pertinent to the dose response relationship to be 0·99 (CI 0.98–1.01) in women and 1.00 (CI 0.99–1.01) in men. Thus, we did not find evidence of a dose response relationship between number of prescriptions for triptans on the risk of stroke, i.e. the RR associated with ever/never use remained unchanged.

The cardiovascular risk factor profile of stroke patients stratified by triptan use is shown in Table 3. Table 4 shows the age-adjusted prevalence of cardiovascular risk factors in stroke patients stratified by use of triptans. The prevalence of cardiovascular risk factors was lower among stroke patients with migraine identified through triptan utilization.

**Table 3:** Risk factors for stroke with respect to triptan use.

		All Non-users (%)	All Users (%)	Men Non-users (%)	Men Users (%)	Women Non-users (%)	Women Users (%)
⁎Alcohol	≤ 14/21 drinks per week	38,302 (78.76)	942 (86.90)	21,846 (76.18)	276 (84.15)	16456 (82.47)	666 (88.10)
	> 14/21 drinks per week	4965 (10.21)	47 (4.34)	4000 (13.95)	23 (7.01)	965 (4.84)	24 (3.17)
	Missing data	5362 (11.03)	95 (8.76)	2,830 (9.87)	29 (8.84)	2532 (12.69)	66 (8.73)
Smoking	Daily smokers	19,502 (40.10)	395 (36.44)	12,101 (42.20)	133 (40.55)	7401 (37.09)	262 (34.66)
	Occasionally smokers	591 (1.22)	15 (1.38)	369 (1.29)	7 (2.13)	222 (1.11)	8 (1.06)
	Former smokers	9664 (19.87)	181 (16.70)	6566 (22.9)	59 (17.99)	3098 (15.53)	122 (16.14)
	Never smokers	13,065 (26.87)	374 (34.50)	6528 (22.76)	97 (29.57)	6537 (32.76)	277 (36.64)
	Missing data	5807 (11.94)	119 (10.98)	3112 (10.85)	32 (9.76)	2695 (13.51)	87 (11.51)
Diabetes	Yes	6329 (13.01)	75 (6.92)	4091 (14.27)	25 (7.62)	2238 (11.22)	50 (6.61)
	No	41,510 (85.36)	997 (91.97)	24,120 (84.11)	298 (90.85)	17,390 (87.15)	699 (92.46)
	Missing data	790 (1.62)	12 (1.11)	465 (1.62)	5 (1.52)	325 (1.63)	7 (0.93)
Atrial fibrillation	Yes	5157 (10.60)	68 (6.27)	3013 (10.51)	21 (6.4)	2144 (10.75)	47 (6.22)
	No	42,493 (87.38)	1000 (92.25)	25,081 (87.46)	300 (91.46)	17,412 (87.27)	700 (92.59)
	Missing data	979 (2.01)	16 (1.48)	582 (2.03)	7 (2.13)	397 (1.99)	9 (1.19)
Prev. myocardial infarct	Yes	3746 (7.70)	36 (3.32)	2740 (9.56)	12 (3.66)	1006 (5.04)	24 (3.17)
	No	43,883 (90.24)	1035 (95.48)	25,349 (88.40)	310 (94.51)	18,534 (92.89)	725 (95.90)
	Missing data	1000 (2.06)	13 (1.20)	587 (2.05)	6 (1.83)	413 (2.07)	7 (0.93)
Hypertension	Yes	22,509 (46.29)	433 (39.94)	13,119 (45.75)	131 (39.94)	9390 (47.06)	302 (39.95)
	No	24,737 (50.87)	621 (57.29)	14,711 (51.30)	186 (56.71)	10,026 (50.25)	435 (57.54)
	Missing data	1383 (2.84)	30 (2.77)	846 (2.95)	11 (3.35)	537 (2.69)	19 (2.51)
Claudication	Yes	1317 (2.71)	13 (1.20)	851 (2.97)	5 (1.52)	466 (2.34)	8 (1.06)
	No	38,761 (79.71)	899 (82.93)	22,798 (79.5)	277 (84.45)	15,963 (80)	622 (82.28)
	Missing data	8551 (17.58)	172 (15.87)	5027 (17.53)	46 (14.02)	3524 (17.66)	126 (16.67)
Total		48,629	1084	28,676	328	19,953	756

⁎ The maximum limit set by the Danish National Board of Health: 14 drinks per week for women and 21 for men.

**Table 4:** Prevalence (%) of risk factors with respect to triptan use adjusted by age (overall and by sex) for patients with stroke.

	All non-users	All users	*p*^b^	Male non-users	Male users	*p*^b^	Female non-users	Female users	*p*^b^
Alcohol^a^	9.28	4.84	< 0.001	13.08	8.61	0.01	4.18	2.97	0.07
Smoking⁎⁎⁎	56.10	47.15	< 0.001	62.72	55.74	0.03	47.78	42.34	0.01
Diabetes	13.87	7.43	< 0.001	15.20	7.84	< 0.001	12.22	7.30	< 0.001
Atrial fibrillation	14.06	10.58	< 0.001	13.58	10.40	0.06	14.87	11.25	< 0.01
Previous myocardial infarct	9.30	4.41	< 0.001	11.58	4.41	< 0.001	6.31	4.33	0.01
Hypertension	51.16	48.49	0.09	49.77	44.34	0.05	53.01	50.87	0.25
Claudication	3.79	1.83	< 0.001	4.12	1.80	< 0.01	3.35	1.85	0.01

a Alcohol consumption over the limit set by the Danish National Board of Health: 14 drinks per week for women and 21 for men.

b Difference in prevalence between triptan users and non-users.

⁎⁎⁎ Only current smokers (i.e. daily and occasional smokers) were considered.

There was no effect modification of oral contraceptive (OC) use on the effect of triptans in subanalysis including women between 25 and 50 years of age: 5.27% of women used triptans while using OC at the same time compared to 5·44% using triptans while not using OC at the same time. Running our model without adjusting for oral contraceptives use risk of stroke among triptan users 25–50 years of age was HR 1·41 (CI 1.27–1.65); adjusting for oral contraceptives risk of stroke among triptan users was HR 1·43 (CI 1.25–1.63).

## 4 Discussion

In this study of the Danish population during nine years of follow-up, based on the concept of identifying migraine through triptan utilization the risk of being hospitalized for stroke was higher among migraineurs. The risk was increased for all strokes and ischemic strokes, while for hemorrhagic stroke risk was increased only in women. The strokes of migraineurs identified through triptan utilization were typically minor strokes while severe strokes were less prevalent.

### 4.1 Stroke Risk Factor Profile

The prevalence of risk factors for stroke among stroke patients who used triptans was markedly lower than among stroke patients who did not use triptans. This might reflect caution by Danish doctors in prescribing triptans for patients with cardiovascular risk factors. The fact that patients with risk factors nevertheless received prescriptions for triptans may indicate a risk–benefit decision. Alternatively, the etiology of strokes in triptan users may differ from that of non-users (with a different risk factor profile) which might suggest a different kind of stroke etiology for triptan users i.e. migraineurs. We had information on the cardiovascular risk factor profiles only for those with stroke; however, the markedly lower age-adjusted prevalence in users of triptans speaks against a higher prevalence of cardiovascular risk factors as the underlying cause of the increased risk for stroke of triptan users. Use of contraceptive pills was the same in users and non-users of triptans and did not influence the effect of triptans. Furthermore, the risk for stroke among triptans users was increased for both sexes.

### 4.2 Cause of Stroke: Triptans vs Migraine

In Denmark, triptans are registered only for the treatment of migraine and cluster headache. As the prevalence of cluster headache is considerably lower than that of migraine, particularly among women (Manzoni and Stovner, 2010), our population of triptan users can be considered as a group of patients with migraine. We found that risk of stroke among triptan users was unrelated to numbers of prescriptions dispensed. Reports on strokes directly related to intake of triptans are rare (Roberto et al., 2015). Thus, overrepresentation of stroke in triptan users in our study seems more likely to be the result of the underlying migraine disorder rather than use of triptans. As a result of the vasoconstricting effect of triptans the question of a possible association between the use of triptans and stroke comes up regularly since the number of users is high (5% of Danish women between 25 and 80 years; 7.5% of Danish women aged 45–50 years). Although the overall risk for stroke in migraineurs in this study was increased only RR 1.07 it was increased RR 1.7 among women aged 25–45 years of age and the incidence of stroke in the triptan using part of the population was not negligible (1.36 per 1000 per year). Our study therefore confirms that any prescription of triptans for headaches should be preceded by careful considerations of the risk for stroke, especially in patients with stroke risk factors.

As the population not prescribed triptans also includes people with migraine who do not need triptans, our study may indicate a risk for stroke only when migraine is severe. If the increased risk for stroke in triptan users reflects an increased risk for stroke among patients with migraine, the risk estimates in our study would be biased towards the null.

### 4.3 Migraine and Stroke

Increased risk for ischemic stroke in migraine have been documented in several meta-analyses (Etminan et al., 2005, Schurks et al., 2009, Spector et al., 2010), as in our study. An increased risk for hemorrhagic stroke was also reported in a recent meta-analysis (Sacco et al., 2013); however, the correlation was found only for women, as the number of men in the analysis did not provide sufficient statistical power. The same was true of our study. Little information is available on the relation between severity of stroke and migraine. In the Women's Health Study, strokes in twenty-two women with migraine with aura were associated significantly more often with a good outcome (Rist et al., 2010). The authors raised the question of whether stroke in migraine patients is different from atherosclerosis and instead specifically involves microvascular phenomena. The results of our study raise the same question. In our study, the 1084 migraineurs identified through triptan utilization were at increased risk for minor strokes but at significantly reduced risk for major strokes in comparison with people not using triptans. The risk for stroke among triptan users was particularly high among women aged 25–40 years, and the risk decreased rapidly with age. This may well be due to the fact that atheromatosis-related risk factors are less frequent in young people, so that strokes with another etiology more easily become significant in statistical analyses. As discussed previously, the markedly lower prevalence of cardiovascular risk factors might also be interpreted as an expression of a different kind of stroke etiology among migraineurs. Our study thus provides further evidence of a biological mechanism for stroke associated with migraine that is different from the mechanisms associated with atheromatosis.

Various causes have been proposed for the higher incidence of stroke in patients with migraine (Kurth and Diener, 2012). Some studies reported a higher incidence of cardiovascular risk factors in patients with migraine, although the elevated risk remained after control for these factors (Kurth and Diener, 2012). In our study prevalence of cardiovascular risk factors were lower among stroke patients with migraine using triptans than among stroke patients at comparable age not using triptans. Several studies indicate that the increased risk for stroke is present only when migraine is associated with aura (Schurks et al., 2009). Studies of brain blood flow during migraine auras have shown focal reduction of cerebral blood flow consistent with ischemia (Olsen et al., 1987, Olsen and Lassen, 1989). Thus, migraine patients with aura may develop ischemia at the microvascular level, which, under some conditions, manifests as a minor stroke. The design of our study did not allow us to distinguish between migraine with and without aura; we were therefore unable to determine whether the increased risk of migraine patients for stroke is associated with both types of migraine or only with migraine with aura. The increased risk for hemorrhagic stroke among migraineurs using triptans in this study and in other studies on migraine and stroke remains to be explained (Kurth and Tzourio, 2013). Suggestions such as altered platelet or endothelial function, use of non-steroidal anti-inflammatory drugs for migraine attacks and structural brain lesions or malformations giving rise to headache mimicking migraine are still hypothetical (Sacco et al., 2013).

### 4.4 Strengths and Limitations

A major strength of this study was the large dataset, with information on age, sex, disposable income, and length of education for the entire Danish population (5.5 million). Furthermore, the personal identification number allowed linkage to the Danish Registry of Prescriptions and to the Danish Stroke Registry, which covers patients hospitalized for stroke in Denmark since 2003. The hospitalization rate according to the registry is high (2.3 per 1000/year) (Andersen et al., 2014) and registration in the Registry has high validity (Wildenschild et al., 2013). The completeness of the data on diabetes, previous myocardial infarction, atrial fibrillation, and hypertension for patients registered with a stroke in this register was > 87%; for alcohol consumption, smoking, and intermittent claudication completeness was > 83%. Information on stroke severity on hospital admission was available for 80% of patients. Therefore, we consider that our study population was representative of both the Danish population of migraineurs prescribed triptans and the Danish population hospitalized for stroke.

A weakness of the study was the lack of information on headache and migraine type nor did we have exact information on the predictive value of using triptan utilization as a proxy for migraine. However, as triptans are licensed in Denmark only for migraine (and cluster headache, as discussed above) and need prescription by an authorized physician, we consider it highly likely that the population with prescriptions for triptans represents a migraine population. The population without prescriptions for triptans includes patients with migraine not using triptans. If, as previously mentioned, the increased risk for stroke in triptan users reflects an increased risk for stroke among patients with migraine, the risk estimates in our study would be biased towards the null.

Users and non-users of triptans may differ according to use of concomitant analgesic agents such as aspirin, paracetamol and NSAID's (non-steroidal anti-inflammatory drugs). As a number of these drugs are delivered over-the-counter they cannot be tracked in the register of prescriptions and cannot be adjusted for in our model. Paracetamol does not influence risk of stroke (García-Poza et al., 2015). Aspirin lowers risk of ischemic stroke but may increase risk of hemorrhagic stroke (Gorelick and Weisman, 2005). While some of the NSAID's appear to increase the risk of stroke, NSAID's as a group do not increase significantly the risk for stroke (García-Poza et al., 2015; Coxib and traditional NSAID Trialists' (CNT) Collaboration, 2013). So even if an effect of these drugs cannot be excluded it will hardly be of a magnitude that it is going to change our conclusions especially regarding ischemic stroke. As > 90% of patients with acute stroke are hospitalized in Denmark (Jørgensen et al., 1992), our observations apply to the majority of stroke patients but only to those who are hospitalized. The Danish Stroke Registry does not include transient ischemic attacks. As the risk of migraineurs prescribed triptans in our study was for minor stroke, inclusion of transient ischemic attacks in the analysis would have been desirable. As another proxy for migraine usage of ergotamine was considered. However, the use of ergotamine in Denmark is so small (0.15% of all Danes have used ergotamine within a 5-year period compared to approx. 4.6% for triptans (i.e. 30 times more frequent)) that it does not serve as a valid comparison.

## 5 In Conclusion

Migraineurs using triptans are at increased risk for both ischemic and hemorrhagic stroke. The excess risk did not seem to be related to the use of triptans as such; instead, our study points to the underlying migraine disease as the cause of the increased risk of stroke among migraineurs. Strokes in migraineurs differed from those of the general stroke population by being less severe and associated with lower prevalence of cardiovascular risk factors. Thus, our study suggests an etiology of stroke in migraineurs different from that of atherosclerotic thromboembolism, probably at the microvascular level. Whatever the cause, stroke is a serious incident in young people undergoing treatment for a disorder that is usually not perceived dangerous. Therefore, our study underlines the necessity of making a careful risk assessment before prescribing triptans for the treatment of headaches. If, as indicated in this study, migraine is associated with an etiology of stroke different from thromboembolism this may have clinical implications for this (although smaller) part of the stroke population in regard to both acute treatment (thrombolytics) and secondary prevention (platelet aggregation inhibitors, statins). More studies are warranted.

## Declaration of Interests

The authors have nothing to disclose.

## Funding

The Helge Peetz og Verner Peetz og hustru Vilma Peetz Foundation (grant no.: 1-24-12-2013).

The authors have not been paid to write this article.

The corresponding author had full access to all the data in the study and had final responsibility for the decision to submit for publication.

## Authors' Contributions

Study concept and design: All authors.

Acquisition, analysis and interpretation of data: All authors.

Drafting of the manuscript: All authors.

Critical revision of the manuscript All authors.

Statistical analysis: Albieri, Andersen.

Obtained funding: Andersen, Olsen.

Study supervision: Andersen, Olsen.

## Role of the Funding Source

The funder had no role in the study design; in the collection, analysis, and interpretation of data; in the writing of the report; and in the decision to submit the paper for publication.
